# Prince of Wales's Hospital Fund

**Published:** 1899-07-22

**Authors:** 


### PRINCE OF WALES'S HOSPITAL FUND.

The special Sunday concerts given in aid of the Prince of Wales's Fund at the Crystal Palace have proved most attractive; two more will be held on Sundays, July 23rd and July 30th, at four o'clock. The London, Brighton, and South Coast, and London, Chatham, and Dover Railways are now issuing special cheap fares to the Palace.

